# Particle size of a new endodontic cement compared to Root MTA and calcium hydroxide

**Published:** 2009-07-06

**Authors:** Elham Soheilipour, Sanam Kheirieh, Majid Madani, Alireza Akbarzadeh Baghban, Saeed Asgary

**Affiliations:** 1 *Researcher, Dental Research Center, Shahid Beheshti University of Medical Sciences, Tehran, Iran*; 2 *Researcher, Iranian Center for Endodontic Research, Shahid Beheshti University of Medical Sciences, Tehran, Iran*; 3 *Researcher, Sympatec Co., Tehran, Iran*; 4 *Department of Biostatistics, Paramedical School, Shahid Beheshti University of Medical Sciences, Tehran, Iran*; 5 *Department of Endodontics, Iranian Center for Endodontic Research, Dental Research Center, Dental School, Shahid Beheshti University of Medical Sciences, Tehran, Iran*

**Keywords:** Calcium hydroxide, CEM cements, New endodontic material, Particle size, Root MTA

## Abstract

**INTRODUCTION:** Particle size and distribution can influence the properties of materials. This study analyzed and compared the particle size of Root MTA, calcium hydroxide (CH), and a new endodontic cement called calcium enriched material (CEM).

**MATERIALS AND METHODS:** The particle size of each material was analyzed three times using 0.05 mg of test material with a particle size analyzer. The particle size distribution ranges, the cumulative percentage and the mean of particle sizes were calculated. One-way ANOVA, Tukey, and Chi-square tests were used for statistical analyses.

**RESULTS:** Results demonstrated that the distribution of particles was dissimilar. Particle mean size in the three different materials was not significantly different. However, the cumulative percentage of CH and CEM cement particles size demonstrated significant difference (P<0.05). Among the various particle size distributions, the particle distribution in the size range of ≤30 μm showed significant difference between materials (P<0.05). Interestingly, the smallest range of particle size belonged to CEM cement.

**CONCLUSION:** The high percentage of small particles found in CEM cement provides desirable properties such as effective seal, good setting time and film thickness in addition to favorable flow and adaptability.

## INTRODUCTION

Different methods have been introduced for particle size analysis for example laser diffraction/image analysis and SEM (1-4) using dry dispersion for dry powders or wet dispersion technique for suspensions/ emulsions. For minute quantities of valuable materials and/or when using a pump that might destroy particles or droplets; the dispersing module CUVETTE is suitable (2).

The distribution of particle size may be an effective method to improve mechanical properties of the different materials (5). Particle size can also influence different characteristics of materials *e.g.* increased surface area (smaller size of particles) can lead to greater dissolution during the setting reaction (6) and a decrease in working time and setting time (7).

Kent and Wilson (8) were one of the pioneers of this type of analyses. Further studies have shown that particle size has little effect on compressive strength (9) and that decrease in particle size leads to increased abrasion resistance of materials (10), higher Compressive Strength (CS) and Diametral Tension Strength (DTS) (1).

A larger mean particle size is also been a contributing factor to the relative weakness of the materials (5,8-11). It has been reported that similar particle sized materials have higher mechanical strength as there is reduced spreading in grid size (1,12). It has been shown that the handling characteristic of cements depends on their particle size and shape (13), moreover the handling characteristics of ceramics and polymers can be improved by particle modification (14,15).

Mineral trioxide aggregate (MTA), a root-end filling material introduced in 1993, is mainly composed of Portland cement (PC) and bismuth oxide. Currently, there are four types of MTA available, including ProRoot MTA and MTA Angelus in gray and white forms (16-18). ‘Root MTA’ is a type of MTA which has been introduced to the Iranian market. Information about the chemical properties of this material is rare. There is, however, one study that analyzed chemical composition of Root MTA and compared it with MTA (19).

The results indicated that the major chemicals of these two materials were not different; they did show significant difference in minor chemicals specially FeO.

MTA has many well known characteristics including biocompatibility and extended setting time (20,21); however it has poor handling and is expensive (22-24).

Komabayashi *et al.* have recently assessed the particle size and shape of CH; most particle size distributions were in the range of 1.0-1.5 µm (25). This study also showed that 74% of particles ranged between 0.5-2.5 µm. They concluded that undissolved particles which penetrate into dentinal tubules may play an important role in antimicrobial effect of CH within dentinal tubules. Moreover, these particles may ionize in and around the tubules and release hydroxide ions; maintaining high pH for prolonged periods (25).

Recently, a new endodontic material in the name of Calcium Enriched Mixture (CEM) cement consisting of different calcium compounds (calcium oxide, calcium phosphate, calcium carbonate, calcium silicate, calcium sulfate and calcium chloride) has been developed (26). In addition to good handling characteristics, CEM cement demonstrated shorter setting times, superior film thickness and flow compared to MTA (26).

There are no articles regarding the particle size of CEM cement; therefore, we aimed to analyze the particle size of CEM cement as well as Root MTA and Calcium Hydroxide.

## MATERIALS AND METHODS

Three types of dental materials including Root MTA (Salami far Dental Supply, Tehran, Iran), CEM cement and Calcium Hydroxide (CH) (lot # K -3825919211, Merck, Darmstadt, Germany) were analyzed in this study. Particle size analyzer model HELOS and disperser CUVETTE with range of measurement between 0.1-3500 µm were used. This analyzer is technically used for emulsions and suspensions through wet technique, in the range of 0.1-3500 µm. CUVETTE includes two 6-mL glass tubes (model SM) for particle size measurements of particles ranged between 0.1-35 µm (with R1 lens) and 50-mL (model US) for particle sizes ranged between 0.25-3500 µm (lens R2-R7). It also includes a mixer for preventing sedimentation, and an ultrasound, for dispersing particles.

Parameters such as reference time, measurement time, time and power of ultrasonic and also the mixture speed were recorded and saved. Fifty mL of ethanol 90% was mixed with 0.05 mg of each sample to acquire a creamy mixture. This mixture was gradually added into the glass tube so that it reached optimal concentration (between 15-27%). Measurements of particle size and dispersion were then performed.

Each mixed material was measured three times to ensure accuracy. This provided three different diagrams that were adapted and then presented as individual data for each experimented material. Regarding the different distribution of particles in test materials which provided only one outcome for each material, the percentages of particle distribution were used as weight variation in weight cases software SPSS; then the mean of particle size was measured for test materials using one-way ANOVA analysis. Tukey HSD test was used for pair comparison. In order to compare the distribution of particles within the various ranges, Chi-square test was used (α=0.05). In order to obtain an improved image of particles, SEM images (TESCAN VEGA, 15kV, Resolution 384×420, mag×2000) were taken for each material.

## RESULTS

**Figure 1:**
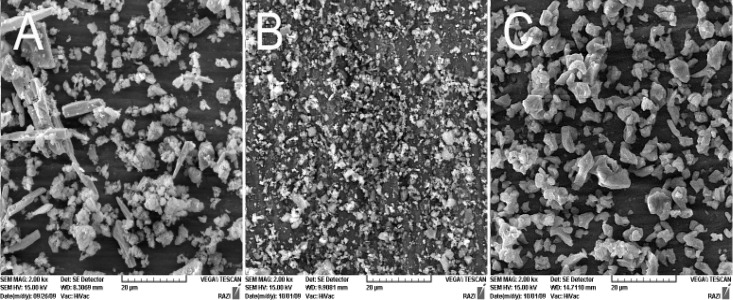
SEM of test materials (×2000): A) Root MTA, B) CEM, C) Ca(OH)_2_

Figure 1 presents the SEM images of test materials (×2000 Mag.). 

**Figure 2:**
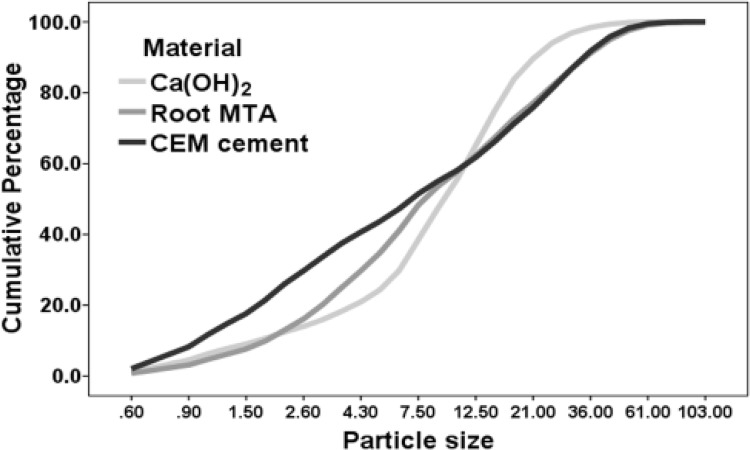
Cumulative percentage of particle size in studied materials

Cumulative percentage related to particle size of the studied materials showed that the distribution of test materials was different (Figure 2).

Though the distribution of particles between CH and CEM cement were significantly different (P<0.05), difference was not observed between Root MTA and CH or Root MTA and CEM cement.

Findings also showed that CH particle sizes were distributed within a narrow range, whereas CEM cement possessed a wider distribution range of particles size. No significant difference was observed between the mean particle sizes of test materials. Table 1 includes descriptive statistical definition, means and standard deviations related to the test materials.

Distribution of particles size <10µm, or between 10-20µm, and 20-30µm was not significantly different.

However, the distribution of particles ≤30μm and >30μm showed significant difference between the three tested materials (P<0.05).

Table 2 demonstrates the distribution of particle sizes between 0.5-30 µm for each of the tested material. CEM cement contained the greatest number of particles within the range of 0.5-2.5μm. Also CEM had the highest percentage within this range (25.7%), while CH and Root MTA’s highest distribution range was between 6.1-15μm (45.0% and 26.3% respectively).

## DISCUSSION

Root MTA, CH, and CEM cement are all water based materials, a hydration reaction occurs when they are mixed with water. Therefore, alcohol was used to produce a suspension for particle detection as well as particle size measurements (22,25,27).

Investigations on dentin tubules in have shown that the density and direction of dentin tubules at the apical root portion of human teeth are irregular (28,29). Generally, the average considered diameter for dentin tubules is between 2-5 µm.

The size of dentin tubules correlates with the particle size of the materials so that particles with smaller size than dentin tubules are able to penetrate through these tubules. This can be an important mechanism for providing a hydraulic three dimensional seal (27,30) and a high local pH (from the ions released) with a slight chance of being reduced by dentin buffering (25), resulting in more effective antibacterial activity. Studies on CEM cement demonstrated that this material is capable of phosphorus and calcium ions release, and, like MTA, contains calcium hydroxide (31,32). These qualities encourage antimicrobial activity (32-34). Similar findings have been previously noted for CH (25).

**Table 1:** Descriptive statistical definition, means and standard deviations related to the test materials

Material	Mean	SD	95% Confidence interval for Mean			Min	Max
			Lower Bound		Upper Bound		
**Ca(OH)**_2_	12.00	9.03	10.21	13.80		0.60	61.00
**Root MTA**	14.91	15.27	11.88	17.95		0.60	103.00
**CEM cement**	14.11	15.18	11.09	17.12		0.60	87.00

**Table 2:** The distribution of particle sizes between 0.5-30 µm for each of the tested material

	Range		material			Total
			Ca(OH) _2_	Root MTA	CEM cement	
**Particle size**	**0.5-2.5**	Count	12	13	26	51
		% within material	12.0	13.1	25.7	17.3
	**2.6-4**	Count	6	12	12	30
		% within material	6.0	12.1	11.9	10.0
	**4.1-6**	Count	11	16	10	37
		% within material	11.0	16.2	9.9	12.3
	**6.1-15**	Count	45	26	19	90
		% within material	45.0	26.3	18.8	30.0
	**15.1-30**	Count	22	19	21	62
		% within material	22.0	19.2	20.8	20.7

The greatest distribution of CEM particle size in our study was within 0.5-2.5 µm range (25.7%) allowing penetration of particles into dentin tubules, and therefore, providing a better seal. This is supported by a previous study that demonstrated superior seal, though not significant, of CEM cement compared with MTA (35,36). The high presence of small size particles in CEM cement may also explain the shorter setting time, better flow and also less film thickness of this dental material which has been demonstrated previously (26).

## CONCLUSION

Small-sized particles of CEM cement were the dominant particles of this material. This enhances its sealing ability and strengthens its physical properties. This new endodontic material is an acceptable alternative for MTA in various clinical applications; however, further investigations are required to determine other properties of this material.
